# Development of a method for the measurement of primary cilia length in 3D

**DOI:** 10.1186/2046-2530-1-11

**Published:** 2012-07-03

**Authors:** Taryn Saggese, Alistair A Young, Chaobo Huang, Kevin Braeckmans, Susan R McGlashan

**Affiliations:** 1 Department of Anatomy with Radiology, Private Bag 92019, University of Auckland, Auckland 1023, New Zealand; 2 Faculty of Pharmaceutical Sciences, Ghent University, Harelbekestraat 72, B-9000 Ghent, Belgium

**Keywords:** 3D microscopy, deconvolution, fluorescent microfibers, Gaussian blurring, kidney cilia, skeletonization

## Abstract

**Background:**

Primary cilia length is an important measure of cell and tissue function. While accurate length measurements can be calculated from cells in 2D culture, measurements in tissue or 3D culture are inherently difficult due to optical distortions. This study uses a novel combination of image processing techniques to rectify optical distortions and accurately measure cilia length from 3D images.

**Methods:**

Point spread functions and experimental resolutions were calculated from subresolution microspheres embedded in 3D agarose gels for both wide-field fluorescence and confocal laser scanning microscopes. The degree of axial smearing and spherical aberration was calculated from *xy*:*xz *diameter ratios of 3D image data sets of 4 μm microspheres that had undergone deconvolution and/or Gaussian blurring. Custom-made 18 and 50 μm fluorescent microfibers were also used as calibration objects to test the suitability of processed image sets for 3D skeletonization. Microfiber length in 2D was first measured to establish an original population mean. Fibers were then embedded in 3D agarose gels to act as ciliary models. 3D image sets of microfibers underwent deconvolution and Gaussian blurring. Length measurements within 1 standard deviation of the original 2D population mean were deemed accurate. Finally, the combined method of deconvolution, Gaussian blurring and skeletonization was compared to previously published methods using images of immunofluorescently labeled renal and chondrocyte primary cilia.

**Results:**

Deconvolution significantly improved contrast and resolution but did not restore the *xy*:*xz *diameter ratio (0.80). Only the additional step of Gaussian blurring equalized *xy *and *xz *resolutions and yielded a diameter ratio of 1.02. Following image processing, skeletonization successfully estimated microfiber boundaries and allowed reliable and repeatable measurement of fiber lengths in 3D. We also found that the previously published method of calculating length from 2D maximum projection images significantly underestimated ciliary length.

**Conclusions:**

This study used commercial and public domain image processing software to rectify a long-standing problem of 3D microscopy. We have shown that a combination of deconvolution and Gaussian blurring rectifies optical distortions inherent in 3D images and allows accurate skeletonization and length measurement of microfibers and primary cilia that are bent or curved in 3D space.

## Background

Primary cilia are small rod-like sensory organelles that protrude from the surface of most mammalian cell types [1]. They range in length from 1 μm in chondrocytes and up to 30 μm in kidney epithelial cells [2-4]. Primary cilia play a role in a vast number of cellular processes including cell cycle control, hedgehog signaling and mechanosensation [1,3,5-8]. Many recent studies have investigated the role of primary cilia length as a means by which the cell can control its sensitivity and fine-tune downstream signaling events [9,10]. Specifically, studies examining primary cilia mechanotransduction in kidney epithelial cells have shown that deflection of the primary cilium results in a Ca^2+ ^signaling event, and that cilia length is proportional to the magnitude of the signaling response [11]. Several previous studies, including our own, have shown that cilia length is sensitive to prolonged periods of mechanical stimulation or insult, for example shortening of either chondrocyte cilia in response to compressive strain [3], endothelial cell cilia in response to flow [12] or kidney cell cilia in response to tubular necrosis [6].

**Figure 1:**
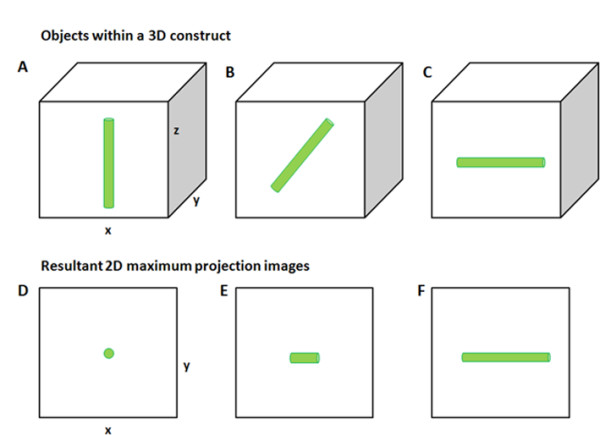
**Effect of 3D orientation on apparent length in 2D maximum projection images**. **(A-C) **3D illustrations of cylindrical objects within a cube of agarose, oriented perpendicular, at 45° and parallel to the plane of focus, respectively. **(D-F) **2D (*xy *plane) maximum projection images of the corresponding 3D objects in (A-C).

Accurate measurement of ciliary length in 3D can be achieved using transmission electron microscopy using either serial sectioning or electron tomography. However, obtaining an entire axonemal profile within a 70 nm section has a probability of 0.5% (1 in 200 cells) [13]. This poses a significant technical challenge and limits the number of cells that can be examined. Therefore, currently, the main method of quantifying primary ciliary length in greater numbers in fixed 2D and 3D cell cultures and tissue sections is by immunofluorescence using antibodies such as acetylated α-tubulin or Arl13b [3,10,14-18]. For *in vitro *cell culture studies, specimens are prepared so that primary cilia are lying flat along the coverslip. Cilia length can then be measured directly from a 2D image using simple line measurement tools associated with the microscope software or a generic image analysis program such as ImageJ http://rsbweb.nih.gov/ij/. However, many studies measure cilia in tissue sections such as kidney [6], in whole mount preparations of zebrafish embryos [7,9] or, as in our studies, chondrocytes cultured in 3D agarose gels [3]. In such 3D preparations, cilia are oriented through several imaging planes within a 3D volume and are bent at random orientations. Due to the 3D nature of these preparations, studies tend to use techniques such as confocal laser scanning or multiphoton microscopy to collect 3D image stacks of primary cilia [3,14,18]. These stacks can be converted to 2D maximum intensity projection images in order to view the entire cilium and make direct length measurements [3]. However, as illustrated in Figure 1, this can lead to inaccurate measurements, since any cilium that does not lie completely parallel to the plane of focus will not appear at full length in the 2D projection image. For a randomly oriented population of cilia, this method would lead to an underestimation of the average cilia length. We and others have previously overcome this problem by only imaging cilia that were approximately 90° to the incident light and ensuring that the maximum *z *depth was ≤ 1.5 μm [3,6,19]. This method results in a biased sample, since only a small subset of cilia are measured. We believe that accurate length measurements can only be obtained from an unbiased population by analyzing the entire 3D volume. However, 3D microscope images contain many optical distortions, such as axial smearing and spherical aberrations, which prevent accurate measurements from being made directly. Several studies have used deconvolution and mathematical modeling of cilia images to accommodate for these optical distortions [18,20]. However, we believe that this approach is suitable for straight cilia and would lead to inaccurate measurement if cilia were twisted or curved within 3D space. Consequently, this study uses a novel combination of established image processing techniques to rectify these optical distortions and accurately measure length from 3D images.

We used custom-made fluorescent microfibers that were embedded in 3D agarose gels representing 'mock' cilia of known lengths to calibrate optical distortions associated with 3D optical microscopy and to test several combinations of established digital imaging processing techniques to measure fiber length within 3D volumes. Finally, we compared our previously published methods with the newly developed method to measure primary cilia of chondrocytes *in vitro *and kidney epithelial cells *in situ*.

## Methods

### Microfibers and microspheres

Model objects of known dimensions were used to validate the ability of digital image-processing techniques to rectify optical distortions and restore object morphology. Two kinds of model objects were used; commercially available fluorescent microspheres were used to optimize and validate deconvolution and Gaussian blurring procedures, while custom made fluorescent microfibers were used to test the accuracy of length measurements.

Microfibers were produced by electrospinning polystyrene solutions containing a fluorophore (coumarin-6). First, a droplet of fluorescent polymer solution was formed at the tip of a needle by surface tension while charge was induced on the droplet surface by an electric field. When the electric field reached a critical value at which the electric force was greater than the surface tension of the droplet, a charged jet was ejected from the tip. While the jet traveled in air, the solvent evaporated, resulting in the deposition of fibers. A rotating collector fitted with a microscope glass slide was used to collect aligned fibers. The fibers were then cut into either 18 or 50 μm lengths by cold ablation using a PALM MicroBeam System which was equipped with a 355 nm pulsed UV-Laser (Version 4.0 AxioVert laser; PALM MicroBeam, Carl Zeiss, Munich, Germany). The fibers were then dry sealed under a coverslip for storage. 2D images of the microfibers were collected to calculate the true length of the fibers prior to further processing. These measurements are herein termed the original fiber population.

The microfibers were resuspended in distilled water and embedded in 3D agarose gel constructs. Agarose constructs were prepared by melting 8% w/v agarose (Sigma-Aldrich, Auckland, New Zealand) in distilled water. Equal volumes of agarose gel and microfibers were combined to yield a final agarose concentration of 4%. The solution was spread out in a thin layer on a microscope slide and set at 4°C for 20 minutes. 3D image stacks of microfibers were acquired from constructs using either wide-field fluorescence (WF) microscopy or confocal laser scanning microscopy (CLSM).

Microspheres with diameters 0.17 μm, 0.2 μm or 4 μm (Invitrogen, Auckland, New Zealand) were diluted 1:1,000 in distilled water and embedded in 3D agarose gels in an identical manner as described for the microfibers. 3D image stacks of the microspheres were acquired under WF or CLSM conditions imaging conditions as appropriate. The differences between experimental WF and CLSM point spread functions (PSFs) are presented in Additional file 1. The effect of imaging depth for agarose specimens is detailed in Additional file 2.

### Immunofluorescent labeling of primary cilia

Wild-type murine chondrocytes embedded in 3D agarose constructs were fixed in 4% paraformaldehyde (PFA) for 30 minutes. Mice were maintained according to approved protocols at the Medical University of South Carolina (AR2646). Heterozygous mice were bred with homozygous Immortomouse mice (*H-2Kb*-tsA58), which harbor a temperature sensitive SV40 large T antigen transgene under the control of an interferon-γ-inducible *H-2Kb *promoter (*H-2Kb*-tsA58) to produce wild-type/Immortomouse compound heterozygous mice. Chondrocytes were isolated from the distal metaphyses of the femurs and proximal metaphyses of the tibiae of 4-day-old mice by digestion with collagenase type II (2 mg/ml) dispersed in Dulbecco's Modified Eagles medium (DMEM; Invitrogen, Auckland, New Zealand) at 37°C for 4 h. Chondrocytes were then cultured in DMEM plus 10% fetal calf serum (FCS; Sigma-Aldrich, Auckland, New Zealand) in the presence of interferon-γ (10 ng/ml; Sigma) at 33°C. To switch off the SV40 gene, cells were cultured in DMEM and 10% FCS at 37°C for 3 days prior to seeding in agarose gels.

Following fixation, chondrocyte agarose constructs were then dehydrated and embedded in paraffin wax using standard histological procedures. Sections 12 μm thick were dewaxed and underwent microwave antigen retrieval by rapid boiling in 0.1 M citrate buffer (pH 6) for 2 × 3 minutes. Paraffin embedded ovine kidney sections (5 μm) were dewaxed and underwent citrate buffer antigen retrieval as above. Ovine kidneys were obtained according to approved protocols at the University of Otago Animal Ethics Committee (AEC88/07). All sections were permeabilized for 5 minutes in 0.5% (v/v) Triton X100 (Global Science & Technology, Auckland, New Zealand), and washed 3 × 5 minutes in phosphate-buffered saline (PBS) + 0.1% (w/v) bovine serum albumin (BSA; Global Science & Technology Ltd, Auckland, New Zealand). Sections were blocked with 5% goat serum (Sigma-Aldrich, Auckland, New Zealand) for 30 minutes, and then incubated with a primary antibody against acetylated α-tubulin, (C3B9; T Sherwin, University of Auckland, Auckland, New Zealand) at 4°C overnight. The sections were washed three times then incubated with goat anti-mouse antibody (Dylight488, 1:500; Jackson ImmunoResearch, West Grove, Pennsylvania, USA) for 2 h at room temperature. Sections were washed and then incubated with Hoechst 33258 (1:500; Sigma-Aldrich, Auckland, New Zealand) for 15 minutes at room temperature. Sections were washed, mounted in Prolong Gold (Invitrogen, Auckland, New Zealand) and sealed with a #1.5 coverslip. Primary cilia were imaged under both WF and CLSM conditions.

### Wide-field fluorescence and confocal laser scanning microscopy

All wide-field fluorescence images were acquired on a Zeiss Axioplan2 upright microscope with motorized *z *stage using a Princeton MicroMax cooled charge-coupled device (CCD) camera controlled with MetaMorph software (Molecular Devices, Sunnyvale, CA, USA). Images were acquired using a 63 × 0.95 numerical aperture (NA) water immersion lens and *xy *pixel and *z *step size were 108 nm and 500 nm for water immersion and 68 nm and 250 nm for oil immersion, respectively. Images were acquired using a filter with 450 to 490 nm excitation and a 520 nm long pass emission. The exposure time for a given specimen was adjusted so that no more than 1 pixel in a 3D image stacks had an intensity of 255.

All CLSM images were acquired using a Leica TC2 SP2 confocal microscope, controlled with Leica Confocal Software version 2.61. Images were acquired with either a 63 × 0.9 NA water immersion lens or a 100 × 1.3 NA oil immersion objective. The *xy *pixel and *z *step size were 66.4 nm and 400 nm for water immersion and 58.1 nm and 250 nm for oil immersion, respectively. Images were acquired using an argon laser with 488 nm excitation and 500 to 560 nm emission. Gain and offset levels were adjusted to ensure that no more than 1 pixel had an intensity of 0 and no more than 1 pixel had an intensity of 255.

### Digital image processing

#### PSF measurements and the calculation of experimental resolutions

Image stacks 8-μm thick of the 0.17 μm and 0.2 μm microspheres embedded in agarose gels were acquired using CLSM and WF microscopy respectively. The image stacks were then analyzed using ImageJ (National Institutes of Health, Bethesda, Maryland, USA); image stacks were converted to an 8-bit format and the maximum pixel intensity in each individual 2D image was measured. The maximum intensity was then plotted against the corresponding *z *position to yield an intensity vs distance plot. The full-width half maximum (FWHM) was then calculated using Sigma plot http://www.sigmaplot.com to yield the *z *resolution. The image stack was then 'resliced' along the *y *axis so that the process could be repeated for intensity vs distance along the *y *axis, subsequently yielding the *xy *resolution.

#### Deconvolution

Blind and non-blind (also termed measured) deconvolution was performed using Huygens Essential deconvolution software (Scientific Volume Imaging, Hilversum, The Netherlands). See Additional file 3 for parameters. The signal to noise ratio (SNR) was estimated by comparing the image quality of a 4 μm microsphere which had undergone blind deconvolution at different SNRs. Once the SNR for each microscope had been determined (SNR = 90 for WF images and SNR = 10 for CLSM), it was used for the deconvolution of all subsequent image stacks. All additional parameters were calculated from the imaging conditions according to the software manual. A table of these parameters is summarized in Additional file 3.

#### Gaussian blurring

Following deconvolution, each 2D image of a subresolution (200 nm) microsphere 3D stack was blurred in the *xy *plane (via a convolution operation) with a Gaussian kernel using ImageJ. This was done to make the resolution isotropic (that is, the same in the *xy *plane as in the *z *direction) in order to enable unbiased length measurement in 3D Kernels of various radii were trialed until the resultant *xy *and *z *experimental resolutions were approximately equalized. The optimized kernel (radius = 3) was then used to blur all other 3D image stacks.

#### Skeletonization

Skeletonization of 3D image stacks was performed using Amira Visualization software (Visage Imaging, Richmond, Victoria, Australia). A binary 3D representation of the object of interest was created within the software. The binary threshold was adjusted manually until the 3D representation visually matched the protection images. The resulting binary object was then eroded, based on Euclidean distance map values until the centerline, that is, the 'skeleton' of the object was produced. The spatial graph function within the Amira software was then used to calculate the length of the skeleton based on the voxel dimensions.

#### 2D maximum projection images

2D maximum intensity projection images were created in ImageJ from 3D WF or CLSM image stacks using the '*z *projection' function.

#### Measurement of object dimensions in 2D

Control microfiber lengths were measured directly from 2D images of the original microfiber population using WF images. The *xy *and *xz *diameter of 4 μm microspheres before and after image processing was measured from binary images of 2D maximum projection images created from the 3D image stacks. Microsphere diameter and microfiber length in 2D was measured using the line measurement tool in ImageJ.

#### Statistical analysis

This study used a calibration approach to assess the accuracy of the new 3D measurement method. The original microsphere diameters were provided from the manufacturer and the original fiber population data was collected from the fibers placed on a microscope slide. Measurements from microspheres and original fibers were expressed as a mean and standard deviation. We considered the measurements obtained from fibers in 3D to be accurate if they fell within 1 standard deviation of the original population mean. To assess the difference between microsphere or microfiber image data following the different types of image processing, a Z test was selected to allow comparisons with the mean and standard deviation of the original populations.

Given that the skeletonization process required a subjective step, we assessed intraobserver variation using three independent observers. Observers were asked to manually threshold, skeletonize and obtain length measurements from the deconvolved and Gaussian blurred 50 μm and 18 μm fiber image sets on two separate days. Length measurements were then compared using paired t tests. The measurements obtained from the three observers were then assessed for interobserver differences using repeated-measures analysis of variance (ANOVA). Intraobserver and interobserver bias was calculated as the mean difference from the original population mean.

As data from primary cilia length measurements were not normally distributed, groups were compared using a Kruskal-Wallis test or Wilcoxon matched pairs t test as appropriate for paired and unpaired data.

For the measurement of primary cilia *in vitro *and *in situ*, the number of cilia that were rejected from image analysis (cilia rejection rate) was calculated as a percentage of resolvable cilia over the total number of cilia that were imaged.

All length and diameter measurements are expressed as a mean ± standard deviation. Statistical tests were performed in GraphPad Prism 5.0 software http://www.graphpad.com. *P *values less than 0.05 were considered statistically significant.

## Results and Discussion

### Microfibers as ciliary models: 2D maximum projection images underestimate microfiber length

**Figure 2:**
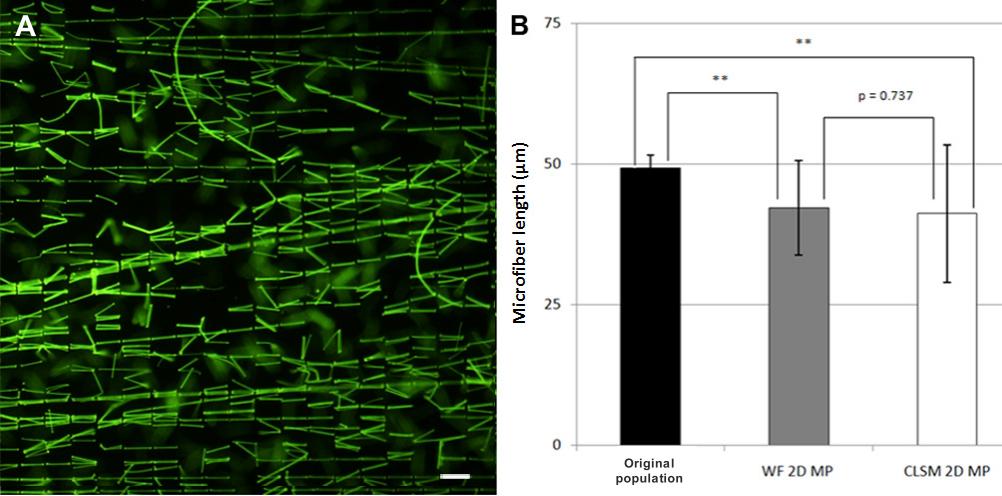
**2D maximum projections (2D MP) significantly underestimate microfiber length in 3D**. **(A) **2D image of the 50 μm microfibers set in their original preparation. **(B) **Mean microfiber (± SD) lengths measured from wide-field fluorescence (WF) or confocal laser scanning microscopy (CLSM) 2D maximum projection images of fibers in 3D constructs. MP measurements from both WF and CLSM data were significantly different from the original population mean. There was no difference in length measurements obtained from MP images of WF and CLSM data (**P ≤ 0.01).

For calibration purposes, we first measured microfiber lengths directly from the two sets of 2D microfiber preparations (50 μm and 18 μm long) using WF microscopy. The measurements obtained are herein termed the original fiber population (Figure 2A). Microfiber length for the longer fibers ranged from 44 to 55 μm, with a mean length of 49.3 ± 2.3 μm (n = 119). The shorter 18 μm fiber set had a range of 11 to 23 μm, with a mean length of 17.9 ± 2.3 μm (n = 318). We then collected 3D image stacks of a sample of the same fibers embedded in 3D agarose constructs using both WF and CLSM to investigate the accuracy of using 2D maximum projection (MP) images for length measurement. Depending on the orientation of the microfibers within 3D, the length in the final 2D MP images of the 50 μm fiber set was significantly reduced, with fiber lengths ranging between 20 μm and 50 μm with a mean length of 41.8 ± 8.9 μm (n = 30). Maximum projection measurements from both WF and CLSM data were significantly different from the original fiber population (*P *= < 0.001 for both WF and CLSM). There was no difference between WF and CLSM data, *P *= 0.737 (Figure 2B). The final length calculation was markedly decreased if any bend or twist was present in a fiber, as is often observed in cilia *in situ*. These results show that 2D maximum projection images do not provide accurate length measurements of objects in 3D preparations.

### Overcoming the optical distortions associated with 3D microscopy: deconvolution combined with Gaussian blurring equalizes lateral and axial resolutions and restores object morphology

**Figure 3:**
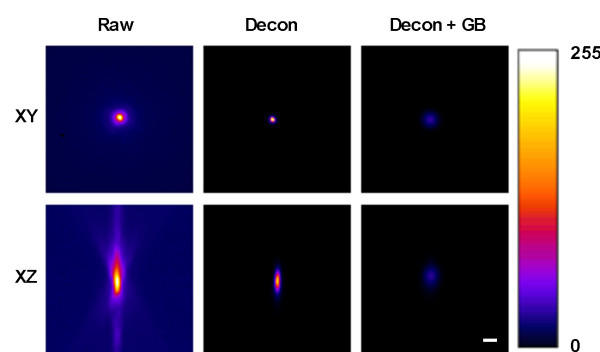
**Creating a spherical point spread function (PSF)**. The discrepancy between axial and lateral resolution is illustrated by the shape of the PSF in the *xy *and *xz *optical planes. The combination of deconvolution (Decon) and Gaussian blurring (GB) equalizes the axial and lateral resolutions. This results in a PSF that is approximately spherical and therefore has the same size and shape in both *xy *and *xz *planes. Scale bar = 1 μm.

Deconvolution, the first image processing technique evaluated, is a digital filtering technique that is specifically designed to rectify the distortions imposed on an image by the optical system arising from the PSF of the system [21-24]. To assess the validity of deconvolution to remove optical distortions, we first collected images from subresolution microspheres in 3D agarose preparations using WF microscopy and a water immersion objective lens. We found that raw (unprocessed) WF 3D images contained large amounts of out-of-focus light in both the *xy *and *xz *planes with significant axial smearing in the *xz *plane (Figure 3). The experimental resolution, as measured from the full-width-half-maximum (FWHM) of an intensity vs distance plot, was 540 nm in the *xy *plane compared with 2,000 nm in the *xz *plane (Table 1). To demonstrate the difference between *xy *and *xz *resolutions, we calculated the *xy:xz *FWHM aspect ratio and found that raw 3D images yielded an *xy:xz *resolution ratio of 0.27 (Table 1). Blind deconvolution produced a significant improvement in contrast and absolute resolution compared to raw images as illustrated in Figure 3 and Table 1. However, the resultant image was still asymmetrical with significant axial smear, yielding an *xy:xz *FWHM aspect ratio of 0.32 (Table 1).

**Table 1:** Making the point spread function (PSF) spherical

	Raw	Deconvolved	Deconvolved and Gaussian blurred
*xy *(nm)	540 ± 76.4	324 ± 76.4	1,080 ± 76.4
*xz *(nm)	2,000 ± 176.8	1,000 ± 176.8	1,000 ± 176.8
Ratio *xy*:*xz*	0.27 ± 0.01	0.32 ± 0.02	1.08 ± 0.02

Full-width-half-maximum (FWHM) measurements of experimental PSFs for raw, deconvolved and deconvolved and Gaussian blurred 3D images (n = 5).

Several methods to overcome axial distortion have been previously reported. Weaver *et al. *used voxel averaging, while Lindig *et al. *decreased the voxel depth by a factor of three to compensate for the effect of axial smearing [25,26]. Soeller and Cannell proposed an alternative solution to overcome the asymmetry of the 3D PSF, by showing that the directional distortions imposed on the 3D image could be removed if the PSF was made effectively spherical. The shape of the optical PSF along the *z *axis can be approximated by a Gaussian distribution; therefore by convolving each individual 2D image in the *xy *plane with a Gaussian kernel, the *xy *resolution would decrease until it approximated the *z *resolution and the resultant PSF would be spherical [27]. The radius of the Gaussian kernel necessary to achieve this correction is proportional to the raw *xy:xz *resolution ratio.

We found when examining 3D images of subresolution microspheres that deconvolution followed by Gaussian blurring created a spherical image with a *xy*:*xz *resolution (FWHM) aspect ratio of 1.08 (Table 1). Gaussian blurring alone (that is, without prior deconvolution) could not be used to create a spherical image, as blurring raw images substantially decreased the contrast, which then prevented binarization. Therefore, the operation could only be carried out on previously deconvolved data sets.

**Figure 4:**
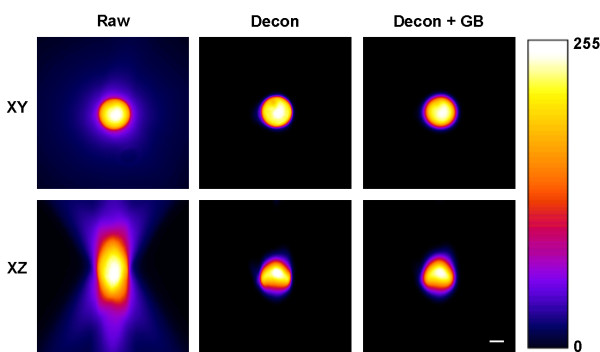
**The combination of deconvolution and Gaussian blurring restores object morphology**. Pseudocolored 2D maximum projection images of a 4 μm microsphere in the *xy *and *xz *plane. The combination of deconvolution (Decon) and Gaussian blurring (GB) rectifies the optical distortion in the images so that the microspheres appear spherical in both *xy *and *xz*. Scale bar = 2 μm.

The combined process of deconvolving and Gaussian blurring was further validated by examining the 3D morphology and diameters of 4 μm microspheres. Deconvolution alone produced a dramatic improvement in the 3D appearance of the microspheres, but overly decreased the diameter of the microspheres in the *xy *plane as measured from binary images (Table 2 and Figure 4). Following Gaussian blurring, there was little change to the appearance of the microsphere in 3D but the *xy *diameter was restored to an accurate value. Consequently, the microsphere diameters were very similar in both planes, giving an *xy*:*xz *diameter ratio of 1.02 (Table 2 and Figure 4). These data show that even though the *xy *resolution was reduced by approximately three times following Gaussian blurring, the processing still achieved accurate diameter measurements in both image planes. The use of these supraresolution (4 μm) microspheres illustrated how the optical pathway distorts the dimensions of objects on a scale with those commonly viewed in life science research such as cells and cellular organelles. Together with data presented in Figure 3 and Table 1, we show that deconvolution combined with Gaussian blurring removes asymmetric distortions within a 3D image.

**Table 2:** Restoration of microsphere morphology

	Raw	Deconvolved	Deconvolved and Gaussian blurred
*xy *(μm)	4.46 ± 0.28	3.16 ± 0.18	4.03 ± 0.31
*xz *(μm)	5.51 ± 0.65	4.01 ± 0.46	4.02 ± 0.46
Ratio *xy*:*xz*	0.82 ± 0.18	0.80 ± 0.11	1.02 ± 0.14

Diameter of 4 μm microspheres (mean ± SD) measured from binary images of *xy *and *xz *2D maximum projections of raw, deconvolved and deconvolved and Gaussian blurred 3D images (n = 17).

### Skeletonization allows measurement of microfiber length in 3D

We then went on to validate the accuracy of skeletonization as a tool to measure length in 3D using the custom-made fluorescent microfibers as models of cilia. Skeletonization is a digital image processing technique that is commonly used to find the medial axis of objects and simplify images of branched structures [28,29]. The skeletonization process begins by creating a 3D reconstruction of the object using a binary mask. The voxels at the edge of the binary object are then successively eroded (based on a Euclidean distance map) until a single row of connected voxels down the center of the object remains, thereby creating a skeleton of the object. Since the dimensions of the voxels are known from the imaging parameters, the length of the object can be directly calculated from this skeleton and the length of curved objects can be measured. Due to the erosion process, skeletonization can decrease the length of any object but only in proportion to the diameter of the object. We believe this was negligible due to the high aspect ratio of the fibers and it did not affect the final length measurements.

**Figure 5:**
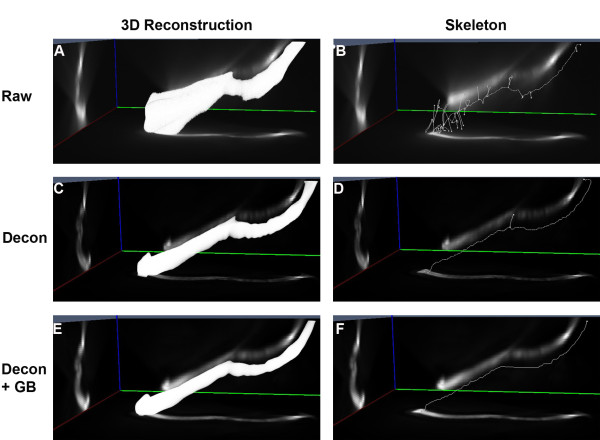
**Skeletonization of microfibers in 3D**. Orthogonal maximum projection images and associated skeletal representation of a microfiber in 3D. **(A, B) **3D binary representation and accompanying skeleton of a raw 3D image set. **(C, D) **Deconvolved 3D data. **(E, F) **Deconvolved and Gaussian blurred data. Images are of a representative fiber from the 50 μm microfiber set.

**Figure 6:**
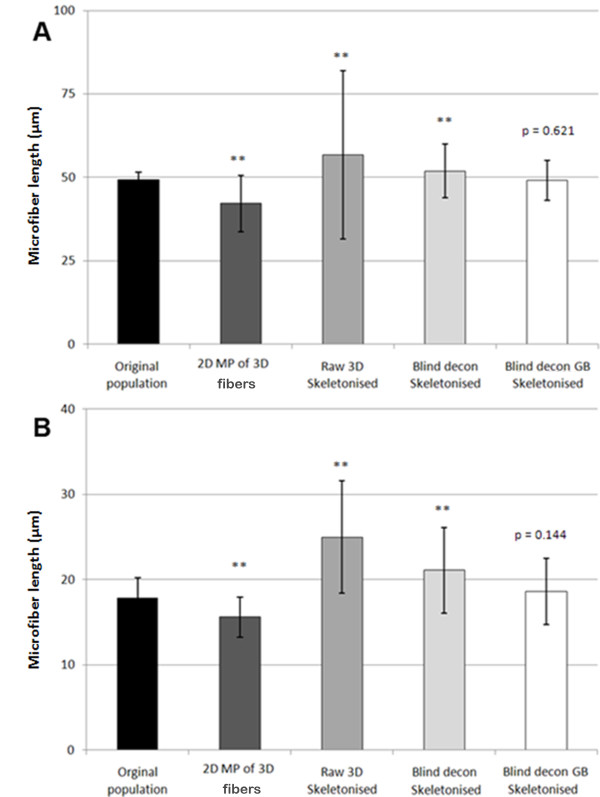
**Comparison of microfiber length by measurement method**. **(A) **The 50 μm fiber set. **(B) **The 18 μm fiber set. For both microfiber sets, only deconvolution combined with Gaussian blurring and skeletonization produced a microfiber length measurement that was not significantly different from the original population. Values represents mean ± SD (**P ≤ 0.01).

We found that the fibers embedded in agarose gel were randomly oriented in 3D and were curved or bent in several different directions. Raw 3D images of microfibers contained prominent optical distortions, visible as blur or flare (Figure 5A, B). The flare around the fiber resulted in secondary structures in the binary 3D reconstruction and consequently appeared as branches in the skeletal representation (Figure 5B). As a result, skeletonization of raw 3D images produced a mean microfiber length of 56.74 ± 25.2 μm and 25.1 ± 6.6 μm for the longer and shorter sets of microfibers, respectively. Deconvolution removed the out-of-focus light and increased contrast in the 3D image, which produced a skeletal representation with fewer branches and a mean microfiber length of 51.9 ± 8.0 μm and 21.1 ± 5.0 μm, respectively. However, only deconvolution combined with Gaussian blurring completely rectified all optical distortions by attenuating the fluorescent intensity along the *z *axis. This yielded an accurate, unbranched, 3D skeletal representation with mean microfiber lengths of 49.1 ± 5.9 μm and 18.6 ± 3.9 μm, respectively (Figure 5F). The 3D images of the microfibers showed that, as with 4 μm microspheres, deconvolution had a dramatic effect on image quality and the additional process of Gaussian blurring did not visually alter the quality of the image (Figure 5). However the subtle adjustment of grayscale values caused by Gaussian blurring over the entire 3D image had a significant effect on the binary image, which in turn, affected the final length calculation. Statistical analysis revealed that only the combination of deconvolution and Gaussian blurring produced a mean fiber length that was not statistically significant different from the fiber length measured from the original fiber population (*P *= 0.6312 and 0.1443 respectively for 50 μm and 18 μm fiber sets; Figure 6A, B). All other methods of measurement produced significantly different lengths from the original population mean length (*P *< 0.001 for all data sets), as shown in Figure 6 and summarized in Additional file 4.

### Repeatability and reproducibility of the 3D measurement method

In order to assess the repeatability and reproducibility of the new 3D method, blind deconvolved and Gaussian blurred 50 μm and 18 μm image sets were measured by three independent observers on two separate occasions. For all observers, the average intraobserver bias was 128 nm and 105 nm for the 50 μm and 18 μm fibers, respectively. Repeatability was assessed using paired t tests to verify if each observer's repeated measurement was significantly different from the first measurement. We found that there was no statistically significant difference between the first and second measurement from each observer suggesting that the method is highly repeatable. The levels of significance for each observer are presented in Table 3.

**Table 3:** Comparison of intraobserver length measurements

Intraobserver	18 μm fibers	50 μm fibers
Observer 1	*P *= 0.540	*P *= 0.628
Observer 2	*P *= 0.133	*P *= 0.797
Observer 3	*P *= 0.972	*P *= 0.758

*P *values from paired t tests comparing each observer's first and second repeated measurement for each microfiber set.

**Figure 7:**
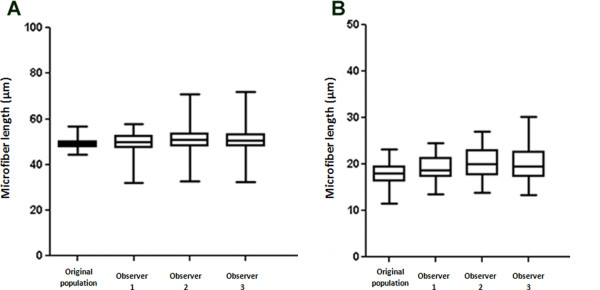
**Interobserver variation**. Microfiber lengths obtained for the original population, compared with the mean measurements made by the three observers using the developed 3D method. **(A) **The 50 μm fiber data set. **(B) **The 18 μm fiber data set.

Interobserver biases were compared using repeated-measures ANOVA and we found that there was no statistical significant difference between measurements from the three observers (*P *= 0.178 and *P *= 0.131 for the 50 μm and 18 μm data sets, respectively). The average biases between observers were 147 nm and 308 nm and are presented in Figure 7. Given the lack of any statistical difference between groups and that the absolute discrepancies fall within the measurement resolution (that is, with the voxel resolution of 108 nm for the *xy *plane and 500 nm for the *z *plane), we believe that the 3D measurement method is within acceptable limits of repeatability and reproducibility.

### Primary cilia in 3D: validation of the 3D measurement method

**Figure 8:**
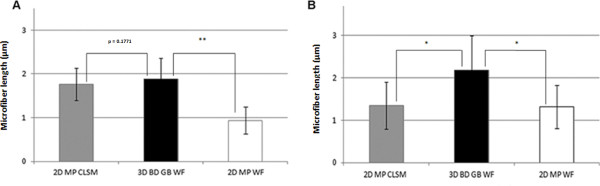
**Primary cilia lengths obtained from 2D and 3D measurement methods**. **(A) **Chondrocyte primary cilia *in vitro *measured using 2D maximum projection images from confocal laser scanning microscopy (CLSM) data, the new 3D method and from 2D maximum projection (MP) images created from the 3D data. (B) Kidney primary cilia *in situ*, measured using 2D and 3D methods. In all instances the 3D measurement method produced a larger mean primary cilia length. (*P ≤ 0.05, **P ≤ 0.01).

Following successful measurement of microfibers, we next assessed the suitability of the 3D measurement method for measuring primary cilia in two different cell types (Figure 8). First, we compared measurements obtained from CLSM maximum projected images (as described in reference 3) with our new 3D method. For chondrocyte cilia, we found no significant difference between length measurements using the two different approaches with a mean length of 1.8 μm (± 0.1) and 1.9 μm (± 0.1), for 2D and 3D methods, respectively (*P *= 0.17; n = 21, Figure 8A). However, the 2D maximum projection method significantly underestimated cilia length in kidney epithelial cells with a mean length of 1.4 μm (± 0.2) compared to 2.2 μm (± 0.2; *P *= 0.001; n = 19) when measured using the 3D method (Figure 8B). This difference was most likely because chondrocyte cilia are relatively short (< 2 μm), and given the resolution limit of an optical microscope, they did not generate a detectable error when measured from 2D projections. However, even though kidney cilia were only slightly longer that the chondrocyte cilia (mean length 2.2 μm), there was a statistically significant difference between 2D and 3D measurements.

Since the previously published method used confocal microscopy, and the new 3D method used wide-field microscopy, we then measured the same cilia from the 3D WF image stacks and compared them with 3D WF images stacks that had been converted into 2D maximum projections. In both chondrocytes and kidney cells, cilia length was significantly shorter when measured in 2D maximum projection images compared to the 3D method (*P *= 0.0001 for chondrocytes, *P *= 0.0007 for kidney cells; Figure 8A, B).

### Limitations of the study

We believe there were two potential sources of variation in this study. First was related to the use of custom-made microfibers. Since fluorescent rod-like calibration objects are not available commercially, we generated two sets of fluorescent polystyrene microfibers to act as models of cilia. However, although we are confident of the fiber length in 2D, we cannot be sure that the fibers did not swell, break or physically distort during processing into agarose gels and believe this is a likely source of the increased variability of microfiber measurements observed with the 3D method compared to original 2D fiber population.

Secondly, the only subjective step in the image processing was the manual thresholding of the 3D image object that was used to create the skeleton. However, we have shown that the method is highly reproducible when tested by three independent observers (see Figure 7).

**Figure 9:**
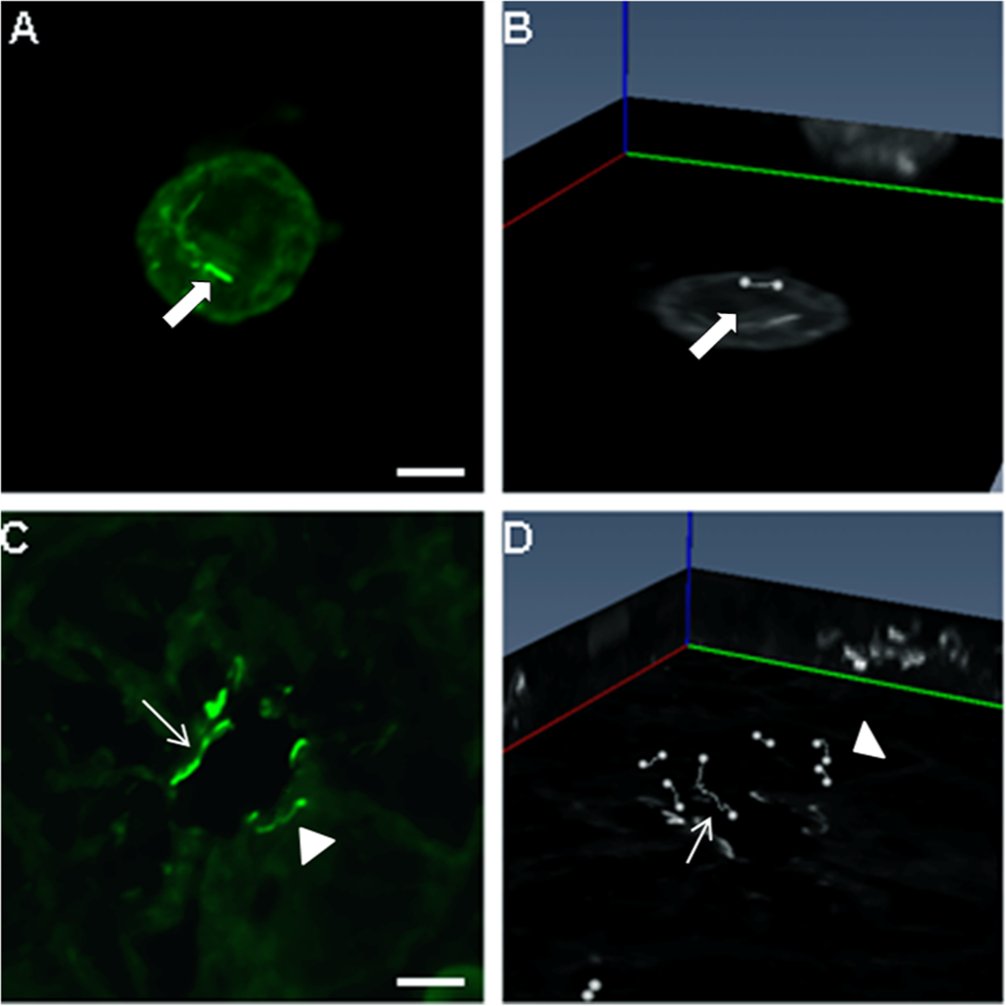
**2D maximum projection (MP) and 3D visualization of primary cilia**. **(A, B) ***In vitro *chondrocyte primary cilia (arrow) labeled with acetylated α-tubulin. (A) 2D maximum projection image, (B) the corresponding 3D projection and skeletal representation. **(C, D) ***In situ *kidney primary cilia viewed as 2D and 3D images. (C, D; arrows) two intertwined cilia that cannot be distinguished in 2D or 3D; (C, D; arrowhead) two cilia that overlap in the 2D MP image that can clearly distinguished in the 3D view. Scale bars = 5 μm.

A further important consideration when using skeletonization is with regard to overlapping or cilia that are in contact. We found that when two single cilia overlapped in 3D space, they appeared as one long cilium in a 2D maximum projection image (Figure 9C, arrowhead). However, these cilia were easily distinguished as individual cilia in 3D (Figure 9D, arrowhead), therefore, the 3D method effectively increased the number of cilia that could be skeletonized within the sample. Specifically, 5% of all cilia examined with the new 3D method were excluded in the analyses because they were physically touching, whereas 21% of cilia were rejected from analyses in the 2D maximum projection images. This is because even if cilia are stacked on top of each other but are separated by several microns in the *z *axis, they would appear as if they are touching in the 2D maximum projection image (Figure 9C). Therefore, as shown in Figure 9, the new method significantly increased the number of cilia that can be measured within a sample.

In addition, many kidney cilia were curved in 3D. Both overlapping and bending would result in additional inaccuracies when measured from 2D maximum projection images. In conjunction with our microfiber data, these results suggest that, unless cilia are shorter than 2 μm and are regularly arranged, accurate length measurements can be achieved using the new 3D method. For objects less than 2 μm in length, it may be sufficient to acquire length measurements directly from 2D maximum projection images.

### Further applications

The work presented in this study was conducted to develop a method for the accurate length measurement of primary cilia *in vitro *and *in situ *in fixed specimens. However the methods used in this paper could be adapted to allow accurate length measurement of objects in 3D under different conditions. Particularly with the development of image analysis software capable of processing 4D image stacks, *in vivo *and dynamic length measurements of primary cilia could be calculated.

## Conclusions

This study used commercial and public domain image processing software to rectify a long-standing problem of 3D microscopy. Our work has shown that 2D maximum projection images significantly underestimate the lengths of randomly oriented microfibers in 3D. Skeletonization enabled length measurement of objects in 3D but only if the images were free of optical distortions. The optical distortions inherent to 3D microscope images were rectified using a combination of deconvolution and Gaussian blurring. The method allowed for accurate length measurements of fibers and primary cilia in 3D with lengths between 2 and 50 μm.

## Competing interests

The authors declare that they have no competing interests.

## Authors' contributions

TS: participated in study design, conducted all microscopy work, performed image and statistical analysis and drafted the manuscript. AAY, KB: participated in study design and helped draft the manuscript. CH: produced microfibers and helped draft the manuscript. SRM: conceived the study, participated in its design and coordination, and drafted the manuscript. All authors read and approved the final manuscript

## Supplementary Material

Additional file 1 **Experimental resolution of wide-field fluorescence (WF) and confocal laser scanning microscopy (CLSM) conditions**. Table of experimental resolutions for WF and CLSM in raw (unprocessed) and deconvolved images and discussion of results. Click here for file

Additional file 2 **The effect of imaging depth on wide-field fluorescence (WF) axial resolution**. Table of experimental resolutions detailing the effect of depth on WF axial resolution due to refractive index mismatch and discussion of the results. Click here for file

Additional file 3 **Deconvolution parameters**. Note on the measurement of the refractive index of agarose and a detailed table of deconvolution parameters. Click here for file

Additional file 4 **Comparison of microfiber length by measurement method**. Tables of 50 μm and 18 μm microfiber data detailing statistical comparison of mean microfiber length by type of measurement method. Click here for file
