# Supplementary material for: Development of a method for the measurement of primary cilia length in 3D
Source: Cilia. 2012 Jul 3;1:11. doi: 10.1186/2046-2530-1-11 (PMC3555708; doi:10.1186/2046-2530-1-11)
Supplement: Additional file 1 — Experimental resolution of wide-field fluorescence (WF) and confocal laser scanning microscopy (CLSM) conditions. Table of experimental resolutions for WF and CLSM in raw (unprocessed) and deconvolved images and discussion of results. [file 2046-2530-1-11-S1.DOC]

**Additional file 1:** The optimization of 3D imaging conditions

*The optimization of 3D imaging conditions: 3D resolution measurements from widefield and confocal laser scanning microscopy*

To determine the resolution which could be achieved on our fluorescent specimens we began by measuring experimental resolutions for both WF and CLSM. Analysis of point spread function (PSF) measurements from sub-resolution microspheres showed that CLSM gave a better 3D resolution than WF microscopy for raw data (Table 1S). In order to improve the resolution of our images the image stacks where filtered using either blind or measured deconvolution. Blind deconvolution uses a theoretically-derived PSF, while measured deconvolution uses an experimentally measured PSF, obtained from images of sub-resolution microspheres (200 nm for WF and 170 nm for CLSM) [23]. We found that blind deconvolution produced a greater or equivalent improvement to 3D resolution (Table 1A). Therefore all subsequent deconvolution procedures were performed using blind deconvolution. Following deconvolution, WF microscopy provided a better final resolution than CLSM (Table 1S). This is most likely due to the low signal-to-noise ratio of the CLSM images resulting in an inaccurate deconvolution operation. Therefore all subsequent experiments were carried out under WF conditions.

**Table 1A.** **Experimental resolutions for WF and CLSM in raw (unprocessed) and deconvolved images**. 3D images stacks of sub-resolution fluorescent microspheres were acquired under water immersion conditions using a 63x NA 0.95 for WF and a 60x NA 0.9 lens for CLSM. Experimental resolutions were measured from the FWHM of intensity vs. distance plots before and after deconvolution ± std dev. (n=5)

|  | **WF Resolution (nm)** | | **CLSM Resolution (nm)** | |
| --- | --- | --- | --- | --- |
|  | **Lateral** | **Axial** | **Lateral** | **Axial** |
| Raw | 540 ± 76.4 | 2000 ± 176.8 | 519 ± 27.9 | 1840 ± 167.3 |
| Blind Deconvolution | 324 ± 76.4 | 1000 ± 176.8 | 385 ± 29.7 | 1320 ± 109.5 |
| Measured Deconvolution | 324 ± 76.4 | 1000 ± 176.8 | 401.6 ± 29.7 | 1320 ± 109.5 |
