# Supplementary material for: Development of a method for the measurement of primary cilia length in 3D
Source: Cilia. 2012 Jul 3;1:11. doi: 10.1186/2046-2530-1-11 (PMC3555708; doi:10.1186/2046-2530-1-11)
Supplement: Additional file 2 — The effect of imaging depth on wide-field fluorescence (WF) axial resolution. Table of experimental resolutions detailing the effect of depth on WF axial resolution due to refractive index mismatch and discussion of the results. [file 2046-2530-1-11-S2.DOC]

**Additional file 2***:*

*Widefield microscopy using water immersion lenses provided optimal resolution at all imaging depths*

An important consideration in 3D microscopy is the effect of refractive index mismatch on 3D resolution [18, 19]. This arises when the refractive index of the objective lens immersion medium differs from the refractive index of the specimen or mounting medium e.g. the combination of oil immersion lens and a water-mounted specimen. The effect is exacerbated by imaging depth [18, 19]. Using experimental PSF measurements, we found that refractive index mismatch adversely affected the axial resolution of oil immersion objectives, with the axial resolution increasing from 1250 nm to 1750 nm when imaging at depths ≥ 20 μm into agarose. Water immersion objectives were not affected by imaging depth (Table 2A). Therefore, all imaging at depths greater than 20 μm was performed with water immersion objectives.

**Table 2A**. **The effect of depth on WF axial resolution due to refractive index mismatch.** Sub-resolution fluorescent microspheres were imaged under WF conditions using either a 63x NA 0.95 water immersion or a 100x NA 1.32 oil immersion objective. Images were acquired at depths of 0, 10 and 20 μm below the coverslip/surface to determine the point at which refractive index mismatch affected resolution. Experimental resolutions were calculated from the FWHM of intensity vs. distance plots. (n= 5 at surface and 3 at each depth)

| **Depth into**  **agarose (μm)** | **Oil lens axial resolution (nm)** | **Water lens axial resolution (nm)** |
| --- | --- | --- |
| Surface | 1250 ± 176.8 | 2000 ± 176.8 |
| 10 | 1250± 0 | 2000 ± 0 |
| 20 | 1750 ± 0 | 2000 ± 0 |
