# Supplementary material for: Development of a method for the measurement of primary cilia length in 3D
Source: Cilia. 2012 Jul 3;1:11. doi: 10.1186/2046-2530-1-11 (PMC3555708; doi:10.1186/2046-2530-1-11)
Supplement: Additional file 3 — Deconvolution parameters. Note on the measurement of the refractive index of agarose and a detailed table of deconvolution parameters. [file 2046-2530-1-11-S3.DOC]

**Additional file 3:** Deconvolution parameters and the measurement of the refractive index of agarose gel

An aliquot of 4% agarose gel was set in a Perspex prism. This prism was positioned on its apex along a horizontal ruler. A laser mounted on the same ruler was used to send a 532 nm wavelength laser beam through the prism, and the output angle was measured using a perpendicular ruler. This output refraction angle was used to calculate the refractive index of the agarose gel. The refractive index of 4% agarose gel was calculated to be 1.34

**Table 3A.** Parameters used for blind deconvolution with Huygens Essential deconvolution software.

| **Parameter** | **WF** | | **CLSM** | |
| --- | --- | --- | --- | --- |
|  | **Water** | **Oil** | **Water** | **Oil** |
| Numerical Aperture | 0.95 | 1.3 | 0.9 | 1.32 |
| Refractive index of lens immersion medium | 1.338 (water) | 1.518 | 1.338 (water) | 1.518 |
| Refractive index of specimen medium | 1.34 (agarose) | 1.46 | 1.34 (agarose) | 1.46 |
| Excitation wavelength (nm) | 470 | 470 | 488 | 488 |
| Emission wavelength (nm) | 515 | 515 | 515 | 515 |
| Back projected pinhole radius (nm) | NA | NA | 330 | 330 |
| Pixel size x (nm) | 108 | 68 | 66.5 | 58.2 |
| Pixel size y (nm) | 108 | 68 | 66.5 | 58.2 |
| Pixel size z (nm) | 300 | 250 | 300 | 200 |
| Vertical mapping function | log | log | log | log |
| Background estimation mode | widefield | widefield | lowest | lowest |
| Area radius | 0.5μm | 0.5μm | 0.5μm | 0.5 μm |
| Maximum iterations | 50 | 50 | 50 | 50 |
| Signal to noise ratio | 90 | 90 | 10 | 10 |
| Quality threshold | 0.01 | 0.01 | 0.01 | 0.01 |
| Iteration mode | optimised | optimised | optimised | optimised |
| Bleaching correction | If possible | If possible | If possible | If possible |
| Brick layout | Auto | Auto | Auto | Auto |
