# Supplementary material for: Development of a method for the measurement of primary cilia length in 3D
Source: Cilia. 2012 Jul 3;1:11. doi: 10.1186/2046-2530-1-11 (PMC3555708; doi:10.1186/2046-2530-1-11)
Supplement: Additional file 4 — Comparison of microfiber length by measurement method. Tables of 50 μm and 18 μm microfiber data detailing statistical comparison of mean microfiber length by type of measurement method. [file 2046-2530-1-11-S4.DOC]

**Additional file 4:** Comparison of microfiber length by measurement method

**Table 4A:** Comparison of measurement method for 50 μm microfibers in 3D

| 50’s | **Original population** | **2D MP** | **Raw skeletonised** | **BD skeletonised** | **BD & GB**  **Skeletonised** |
| --- | --- | --- | --- | --- | --- |
| **Mean ± std dev** | 49.3 ± 2.3 | 42.2 ± 8.4 | 56.74 ± 25.2 | 51.9 ± 8.0 | 49.1 ± 5.9 |
| **n** | 119 | 30 | 30 | 30 | 30 |
| **x-μ** |  | -7.1 | 7.44 | 2.6 | -0.2 |
| **Z statistic** |  | 16.9 | 17.7 | 6.2 | 0.48 |
| **P value** |  | <0.001 | <0.001 | <0.001 | **0.6213** |

**Table 5A**: Comparison of measurement method for 18μm microfibers in 3D

| 18’s | **Original population** | **2D MP** | **Raw skeletonised** | **BD skeletonised** | **BD & GB**  **Skeletonise** |
| --- | --- | --- | --- | --- | --- |
| **Mean ± std dev** | 17.9 ± 2.3 | 15.6 ± 2.3 | 25.1 ± 6.6 | 21.1 ± 5.0 | 18.6 ± 3.9 |
| **n** | 318 | 23 | 23 | 23 | 23 |
| **x-μ** |  | 2.3 | 7.2 | 3.2 | 0.7 |
| **Z statistic** |  | 4.8 | 15 | 6.7 | 1.46 |
| **P value** |  | <0.001 | <0.001 | <0.001 | **0.1443** |
